# Supplementary material for: MaxEnt’s parameter configuration and small samples: are we paying attention to recommendations? A systematic review
Source: PeerJ. 2017 Mar 14;5:e3093. doi: 10.7717/peerj.3093 (PMC5354112; doi:10.7717/peerj.3093)
Supplement: Supplemental Information 1 [file peerj-05-3093-s001.docx]

**S1**. List of databases of the “Web of Knowledge” engine used for the literature search process.

| Databases |  |
| --- | --- |
|  |  |
| Web of Science^TM^ Core Collection (1981-present) | |
| BIOSIS Citation Index^SM^ (1926-present) | |
| Current Contents Connect® (1998-present) | |
| Inspec® (1898-present) | |
| KCI-Korean Journal Database (1980-present) | |
| MEDLINE® (1950-present) | |
| Russian Science Citation Index (2005-present) | |
| SciELO Citation Index (1997-present) | |
| Zoological Record® (1864-present) | |
