# Supplementary material for: MaxEnt’s parameter configuration and small samples: are we paying attention to recommendations? A systematic review
Source: PeerJ. 2017 Mar 14;5:e3093. doi: 10.7717/peerj.3093 (PMC5354112; doi:10.7717/peerj.3093)
Supplement: Supplemental Information 3 [file peerj-05-3093-s003.docx]

**S3**. Methodology used for analyzing the difference on modelling results when using Maxent’s default parameters and optimal parameters combination.

**Software**

We used three different free software packages to perform all the analyses and quantifications. The software packages and their description are the following:

1. Maximum Entropy Modeling (MaxEnt): Maxent is a free software package that uses a maximum entropy algorithm to predict species distribution using species presence and environmental data created by Phillips *et al.* (2006). Available for download from this link http://www.cs.princeton.edu/~schapire/MaxEnt.

2. Environmental niche models tools (ENMTools): ENMtools is a set of different tools to make quantitative comparisons of niche models created by Warren *et al.* (2011). Some of tools included are: niche similarity testing, correlation analysis, model selection analysis. Available for download from this link <http://www.danwarren.net/enmtools/builds/ENMTools_1.4.4.zip>

3. Quantum Source Geographic Information System (QGIS): QGIS is an open source Geographic Information System (GIS) created by the open source geospatial foundation (OSGeo). QGIS allows the visualization, manage, edition and analysis of geographical data. It also included a powerful tool that allows the user to compose printable maps. Available for download from this link <http://www.qgis.com>.

1. Raster similarity analysis (MCK): Map comparison kit (MCK) is free software tool that allows comparing a pair of raster maps in terms of their similarity; using a range of different algorithm. It includes analysis methods as Kappa, percentage of agreement and newly developed approaches as the Fuzzy Kappa statistic. The software was created by Research Institute for Knowledge Systems. Available for download from this link

http://mck.riks.nl/ downloads

**Methodology**

We used MaxEnt software version 3.3.3k (Phillips *et al.* 2006; Phillips & Dudík 2008) to model present and future potential habitat suitability of *B. miersii* and *P. splendens*. Also, we used 19 bioclimatic layers generated from the interpolation of climatic data compiled around the world with a resolution of ~1 Km^2^ (Hijmans *et al.* 2005). We aggregated species presence points to match climatic data resolution avoiding pseudo replication.

To ensure the quality of the final habitat suitability models and to reduce potential over-parameterization (Merow *et al.* 2013) we performed a Pearson correlation analysis of the 19 bioclimatic variables using for that the software ENMTools version 1.4.4 (Warren *et al.* 2011). As suggested by previous studies (Kumar & Stohlgren 2009), all variables with correlations larger than 0.8 were evaluated to retain only those more relevant for the species ecology.

To select the best model parameters we compared different models with a combination of the “feature class” and “regularization multiplier”. MaxEnt provides different types of restrictions (“feature class”) in the modelling stage such as lineal (L), quadratic (Q), product (P), threshold (T), and hinge (H). We used all the possible combinations of these features (12 combinations). The used regularization multiplier values were based on Warren and Seifert (2011) and Shcheglovitova & Anderson (2013): 1, 2, 5, 10, 15, and 20. Combining features classes and regularization multipliers, we assessed a total of 72 models for each case study, plus the default auto-feature.

We compared all the generated models by utilizing the corrected Akaike information criterion (AICc) available in the software ENMTOOLS version 1.4.4 (Warren *et al.* 2011). The process of choosing the best model using AICc has some limitations that can have an impact on the final results. Results of simple model selection implemented by ENMTOOLS should be carefully interpreted because uncertainties of each parameter values may not be fully incorporated for calculation of AICc. AICc can excessively penalize overparametrization which can lead to choosing the wrong model (Hastie *et al.* 2009). Despite this fact, simulation have shown that AICc perform fairly well (Warren & Seifert 2011, Warren *et al.*2014).

To generate the final model for less than 25 samples we used the bootstrapping option with a number of replications equal to the number of samples. In cases were there more than 25 samples we used a random sample of 70% of the presence points, while the 30% remaining points were used to validate the model.

We produced three types of output maps for our analysis: (1) Logistic output maps, corresponding to the output in continuous pixel values for the entire modeled area, which were used as the input for estimating the spatial correlation between models. (2) Threshold logistic maps, corresponding to the output in continuous pixel values only for the areas with pixel values above the selected thresholds, which were used to calculate the fuzzy kappa statics using the Map Comparison Kit (MCK) based on Mestre et al. 2015. (3) Binary maps, corresponding to the areas defined as suitable and non-suitable after applying the selected threshold value, which were used to perform the area-based analyses.

For threshold logistic and binary maps, we used the “10 percentile training presence logistic threshold” as it is among the most commonly used thresholds for creating suitability maps for species distribution with MaxEnt (Contreras-Medina *et al.* 2010, Vale *et al.* 2014). All output maps were created using the software Qgis 2.10 Pisa ([www.qgis.com](http://www.qgis.com)). For the map comparison analysis we used the “Numerical Fuzzy Kappa” analysis provided in the software Map Comparison Kit 3.2.3 (MCK) (Visser & De Nijs 2006).

**References**

Contreras-Medina, R., Luna-VegA, I. & Ríos-Muñoz, C.A. (2010) Distribución de Taxus globosa (Taxaceae) en México: Modelos ecológicos de nicho, efectos del cambio del uso de suelo y conservación. *Revista chilena de historia natural*, **83**, 421-433.

Hastie, T., Tibshirani, R. & Friedman, J. (2009) *The elements of statistical learning : data mining, inference and prediction*, 2nd ed. edn. New York, N.Y. : Springer, New York, USA.

Hijmans, R.J., Cameron, S.E., Parra, J.L., Jones, P.G. & Jarvis, A. (2005) Very high resolution interpolated climate surfaces for global land areas. *International Journal of Climatology*, **25**, 1965-1978.

Kumar S., Stohlgren T.J. (2009) MaxEnt modeling for predicting suitable habitat for threatened and endangered tree *Canacomyrica monticola* in New Caledonia. *Journal of Ecology and Natural Environment* **1**, 94–98

Merow, C., Smith, M.J. & Silander, J.A. (2013) A practical guide to MaxEnt for modeling species’ distributions: what it does, and why inputs and settings matter. *Ecography*, **36**, 1058-1069.

Mestre, F., Pita, R., Paupério, J., Martins, F.M.S., Alves, P.C., Mira, A. & Beja, P. (2015) Combining distribution modelling and non-invasive genetics to improve range shift forecasting. Ecological Modelling, **297**, 171–179.

Phillips, S.J. & Dudík, M. (2008) Modeling of species distributions with Maxent: new extensions and a comprehensive evaluation. *Ecography*, **31**, 161-175.

Phillips, S.J., Anderson, R.P. & Schapire, R.E. (2006) Maximum entropy modeling of species geographic distributions. *Ecological Modelling*, **190**, 231-259.

Shcheglovitova, M. & Anderson, R.P. (2013) Estimating optimal complexity for ecological niche models: A jackknife approach for species with small sample sizes. *Ecological Modelling*, **269**, 9-17.

Vale, C.G., Tarroso, P. & Brito, J.C. (2014) Predicting species distribution at range margins: testing the effects of study area extent, resolution and threshold selection in the Sahara–Sahel transition zone. *Diversity and Distributions*, **20**, 20-33.

Visser, H., de Nijs, T., 2006. The Map Comparison Kit. Environ. Model. Softw. 21, 346–358.

Warren, D.L. & Seifert, S.N. (2011) Ecological niche modeling in Maxent: the importance of model complexity and the performance of model selection criteria. *Ecological Applications*, **21**, 335-342.

Warren, D.L., Wright, A.N., Seifert, S.N. & Shaffer, H.B. (2014) Incorporating model complexity and spatial sampling bias into ecological niche models of climate change risks faced by 90 California vertebrate species of concern. *Diversity and Distributions*, **20**, 334-343.
