# Supplementary material for: MaxEnt’s parameter configuration and small samples: are we paying attention to recommendations? A systematic review
Source: PeerJ. 2017 Mar 14;5:e3093. doi: 10.7717/peerj.3093 (PMC5354112; doi:10.7717/peerj.3093)
Supplement: Supplemental Information 4 [file peerj-05-3093-s004.docx]

**Map comparison results**

**
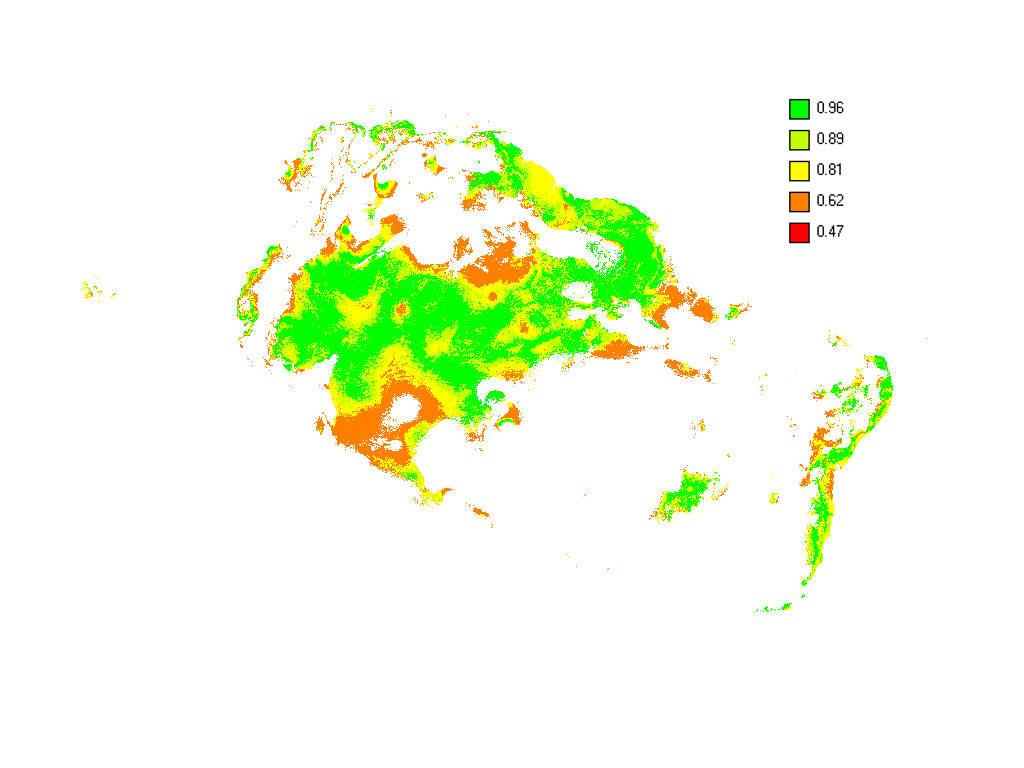
**

**S4.1.** Numerical fuzzy similarity map results for Aguilar et al. 2015. The fuzzy similarity map and corresponding Fuzzy Kappa statistic, using the ‘Numerical Fuzzy kappa algorithm’, a value of 1.0 (dark green) means total similarity and a value of 0.0 total dissimilarity. Values in orange (around 0.6) point to some similarity in both maps.

**
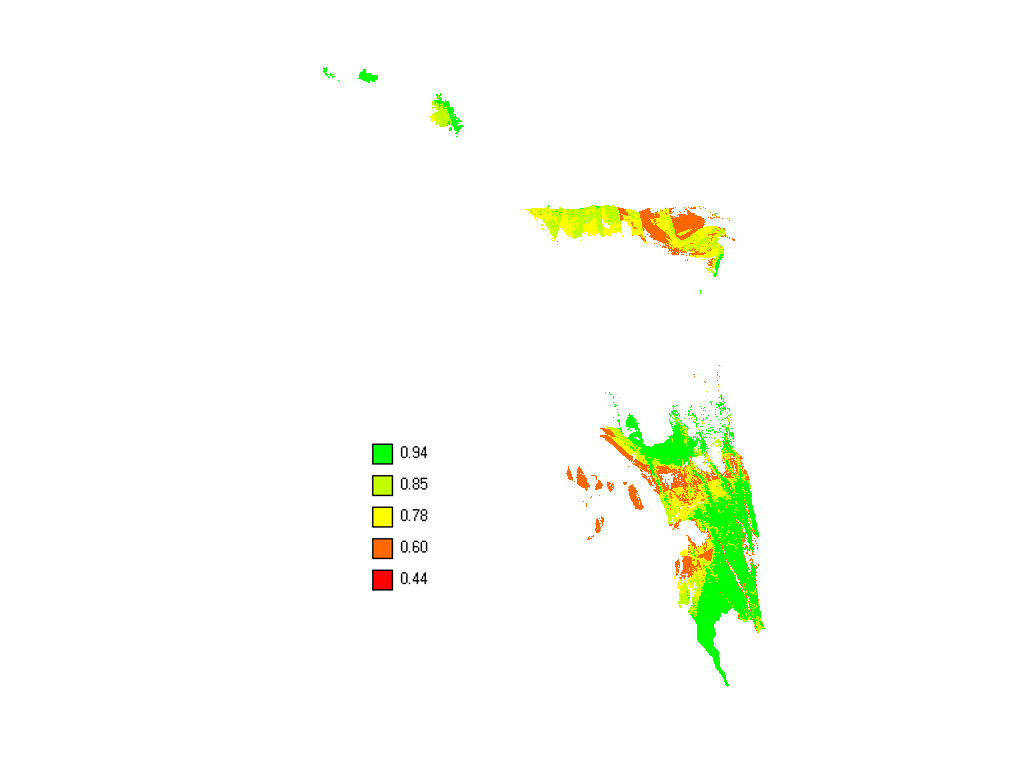
**

**S4.2.** Numerical fuzzy similarity map results for Alamgir et al. 2015. The fuzzy similarity map and corresponding Fuzzy Kappa statistic, using the ‘Numerical Fuzzy kappa algorithm’, a value of 1.0 (dark green) means total similarity and a value of 0.0 total dissimilarity. Values in orange (around 0.6) point to some similarity in both maps.

**
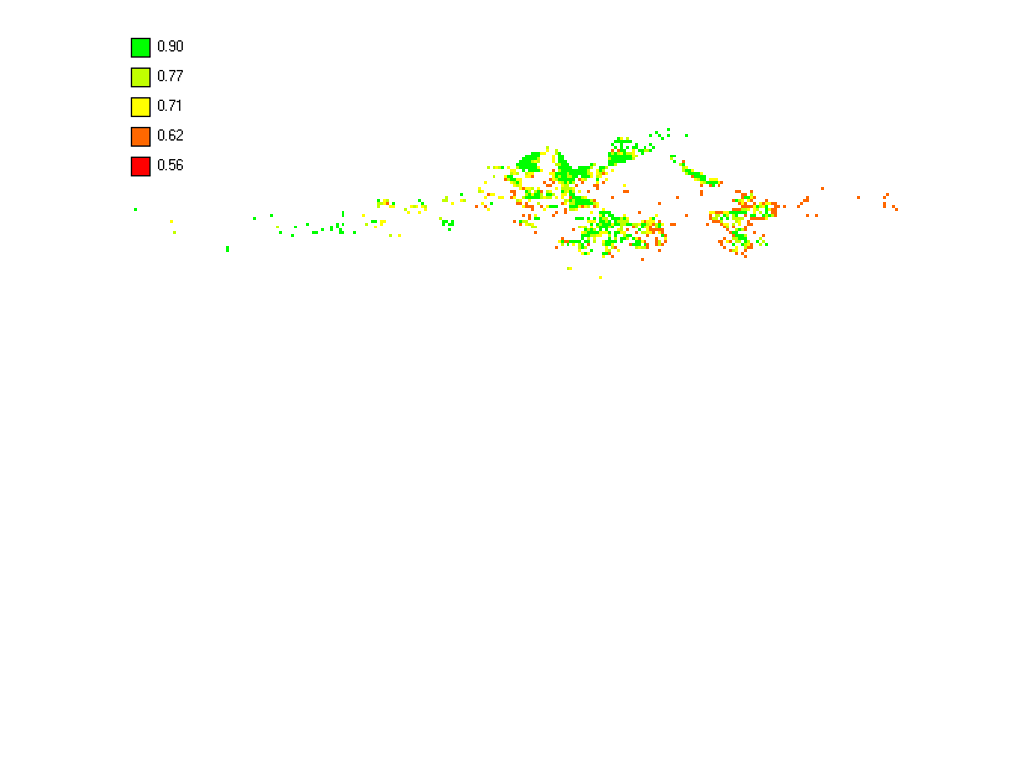
**

**S4.3.** Numerical fuzzy similarity map results for Alfaro-Saiz et al. 2015. The fuzzy similarity map and corresponding Fuzzy Kappa statistic, using the ‘Numerical Fuzzy kappa algorithm’, a value of 1.0 (dark green) means total similarity and a value of 0.0 total dissimilarity. Values in orange (around 0.6) point to some similarity in both maps.


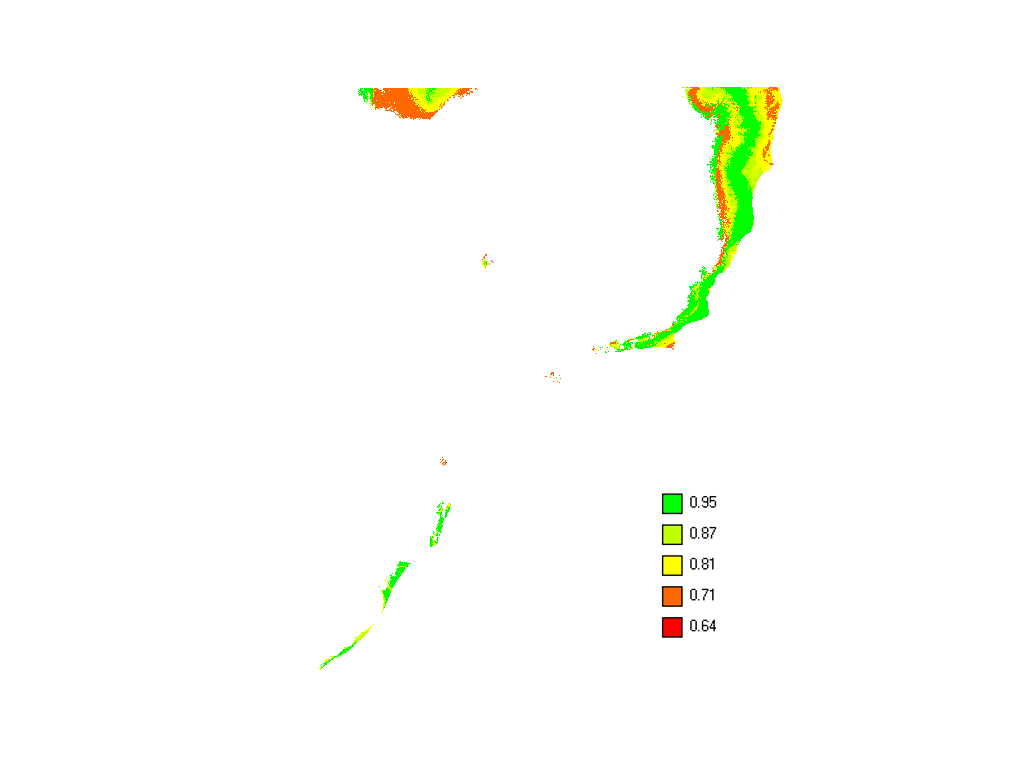


**S4.4.** Numerical fuzzy similarity map results for Carvalho et al. 2015. The fuzzy similarity map and corresponding Fuzzy Kappa statistic, using the ‘Numerical Fuzzy kappa algorithm’, a value of 1.0 (dark green) means total similarity and a value of 0.0 total dissimilarity. Values in red (around 0.64) point to some similarity in both maps.


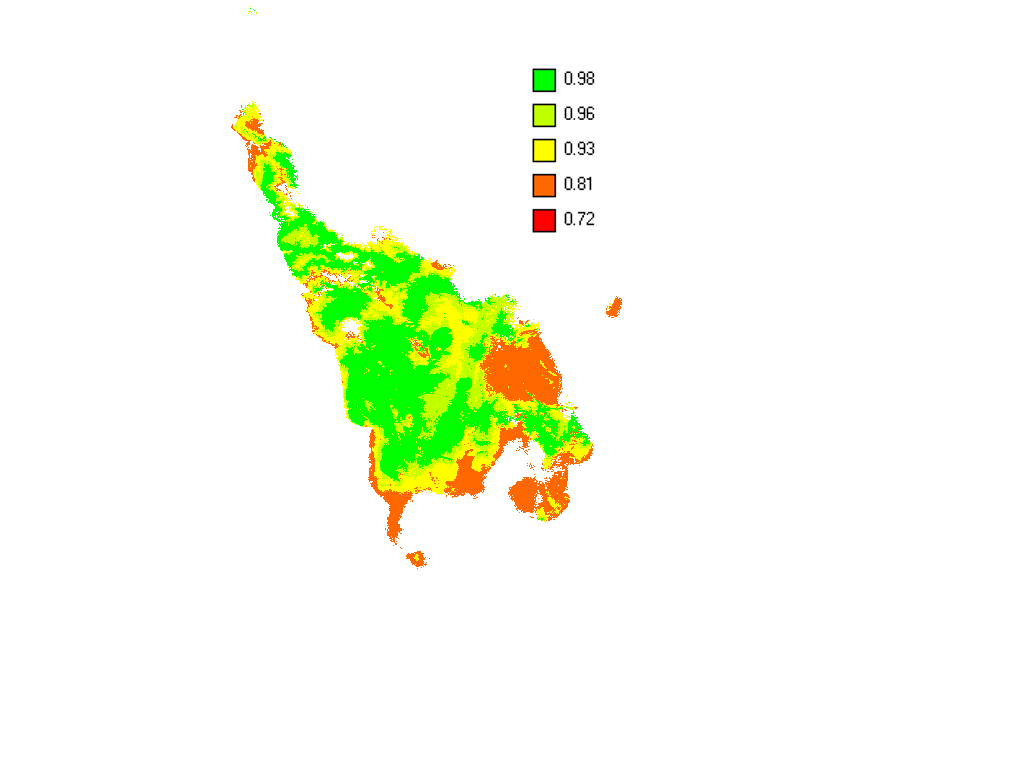


**S4.5.** Numerical fuzzy similarity map results for Chetan et al. 2014. The fuzzy similarity map and corresponding Fuzzy Kappa statistic, using the ‘Numerical Fuzzy kappa algorithm’, a value of 1.0 (dark green) means total similarity and a value of 0.0 total dissimilarity. Values in red (around 0.72) point to some similarity in both maps.

**
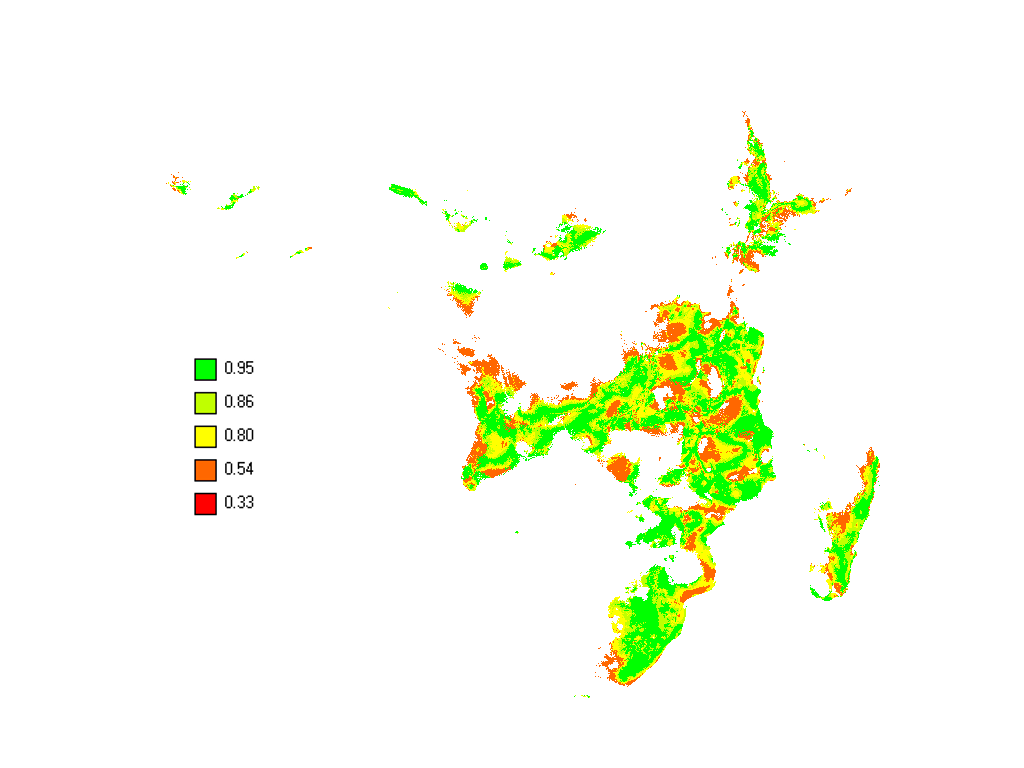
**

**S4.6.** Numerical fuzzy similarity map results for Chlond et al. 2015. The fuzzy similarity map and corresponding Fuzzy Kappa statistic, using the ‘Numerical Fuzzy kappa algorithm’, a value of 1.0 (dark green) means total similarity and a value of 0.0 total dissimilarity. Values in orange (around 0.54) point to some similarity in both maps.

**
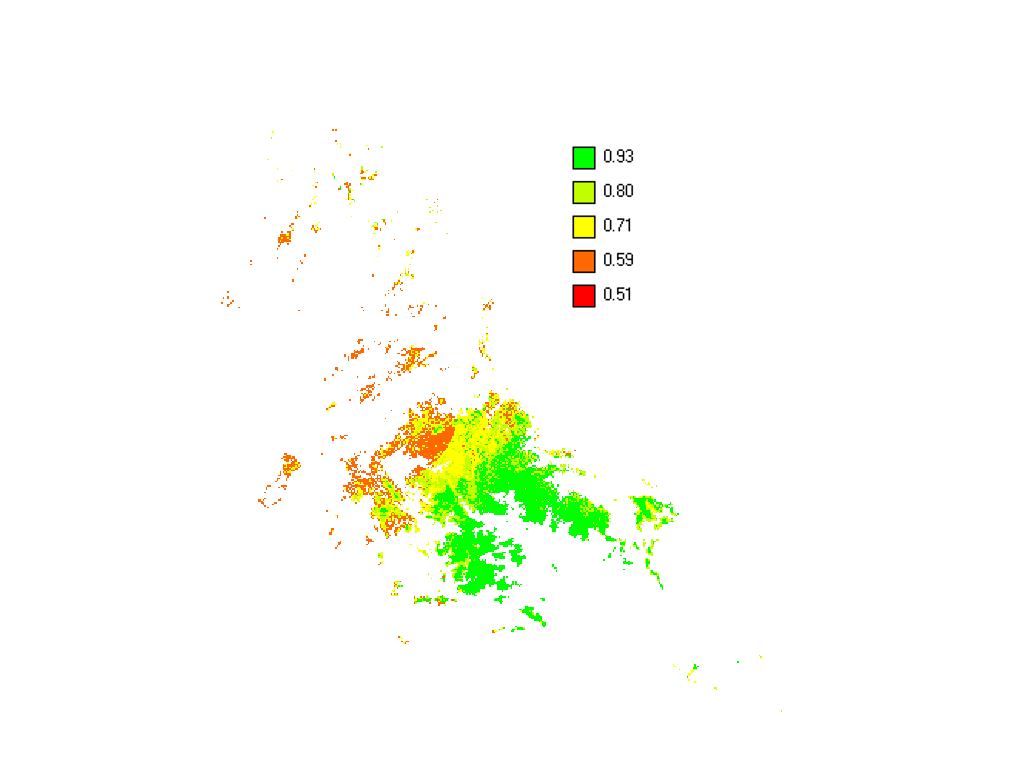
**

**S4.7.** Numerical fuzzy similarity map results for Chunco et al. 2013. The fuzzy similarity map and corresponding Fuzzy Kappa statistic, using the ‘Numerical Fuzzy kappa algorithm’, a value of 1.0 (dark green) means total similarity and a value of 0.0 total dissimilarity. Values in red (around 0.51) point to some similarity in both maps.


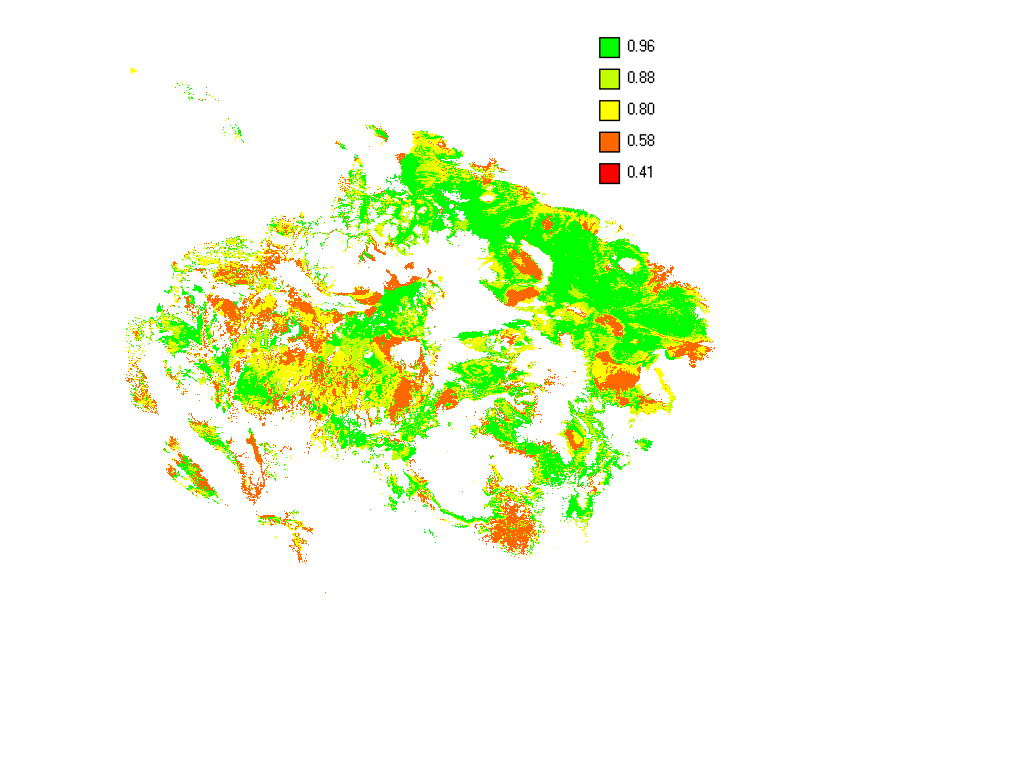


**S4.8.** Numerical fuzzy similarity map results for Confliti et al. 2015. The fuzzy similarity map and corresponding Fuzzy Kappa statistic, using the ‘Numerical Fuzzy kappa algorithm’, a value of 1.0 (dark green) means total similarity and a value of 0.0 total dissimilarity. Values in orange (around 0.58) point to some similarity in both maps.

**
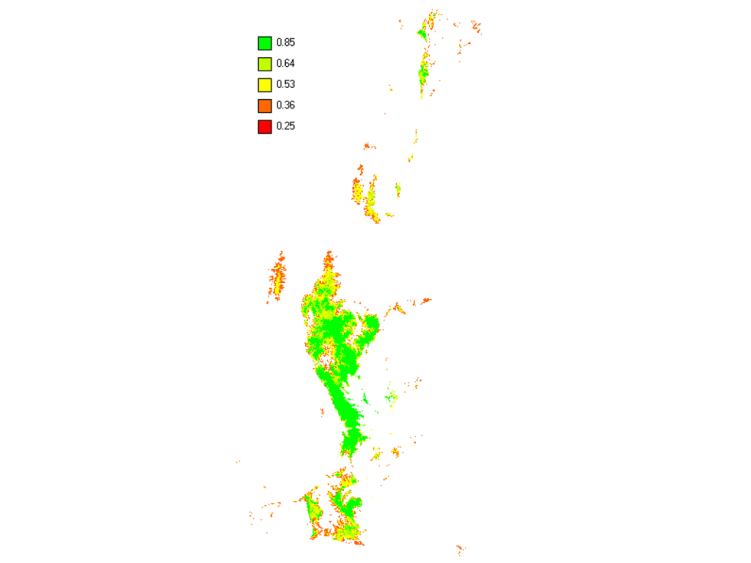
**

**S4.9.** Numerical fuzzy similarity map results for De Castro et al. 2014. The fuzzy similarity map and corresponding Fuzzy Kappa statistic, using the ‘Numerical Fuzzy kappa algorithm’, a value of 1.0 (dark green) means total similarity and a value of 0.0 total dissimilarity. Values in yellow (around 0.53) point to some similarity in both maps.


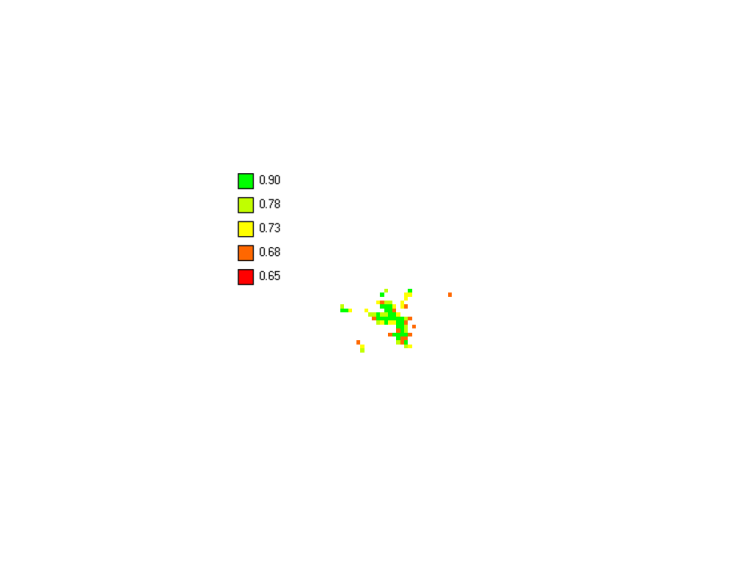


**S4.10.** Numerical fuzzy similarity map results for Fois et al. 2014. The fuzzy similarity map and corresponding Fuzzy Kappa statistic, using the ‘Numerical Fuzzy kappa algorithm’, a value of 1.0 (dark green) means total similarity and a value of 0.0 total dissimilarity. Values in red (around 0.65) point to some similarity in both maps.


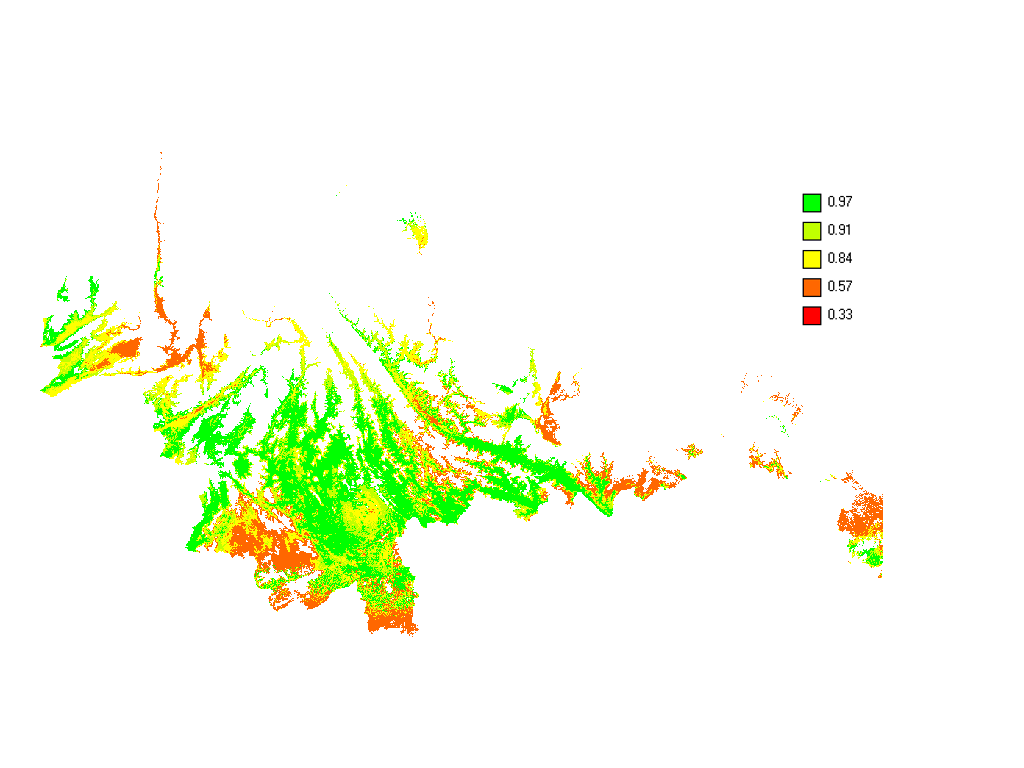


**S4.11.** Numerical fuzzy similarity map results for Hu et al. 2015. The fuzzy similarity map and corresponding Fuzzy Kappa statistic, using the ‘Numerical Fuzzy kappa algorithm’, a value of 1.0 (dark green) means total similarity and a value of 0.0 total dissimilarity. Values in red (around 0.65) point to some similarity in both maps.

**
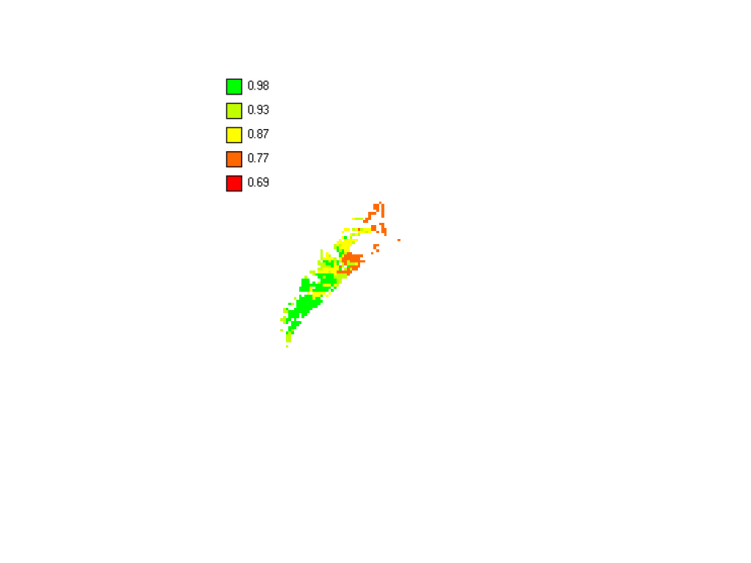
**

**S4.12.** Numerical fuzzy similarity map results for Meyer et al. 2014. The fuzzy similarity map and corresponding Fuzzy Kappa statistic, using the ‘Numerical Fuzzy kappa algorithm’, a value of 1.0 (dark green) means total similarity and a value of 0.0 total dissimilarity. Values in red (around 0.69) point to some similarity in both maps.


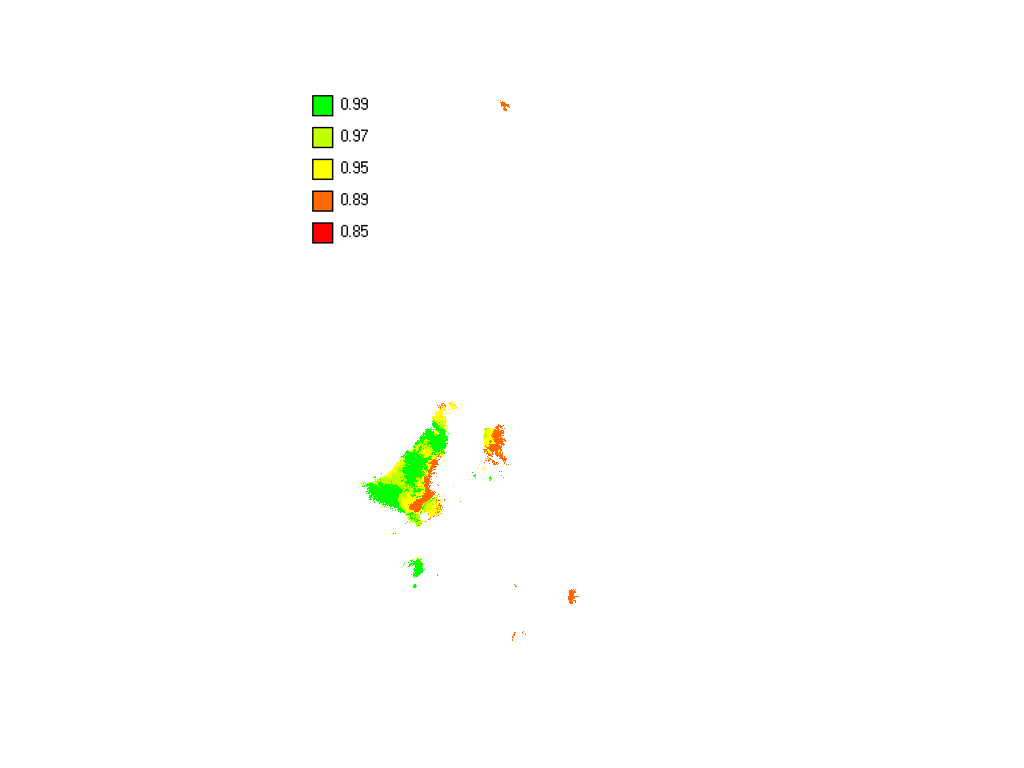


**S4.13.** Numerical fuzzy similarity map results for Mweya et al. 2013. The fuzzy similarity map and corresponding Fuzzy Kappa statistic, using the ‘Numerical Fuzzy kappa algorithm’, a value of 1.0 (dark green) means total similarity and a value of 0.0 total dissimilarity. Values in red (around 0.85) point to moderate similarity in both maps.

**
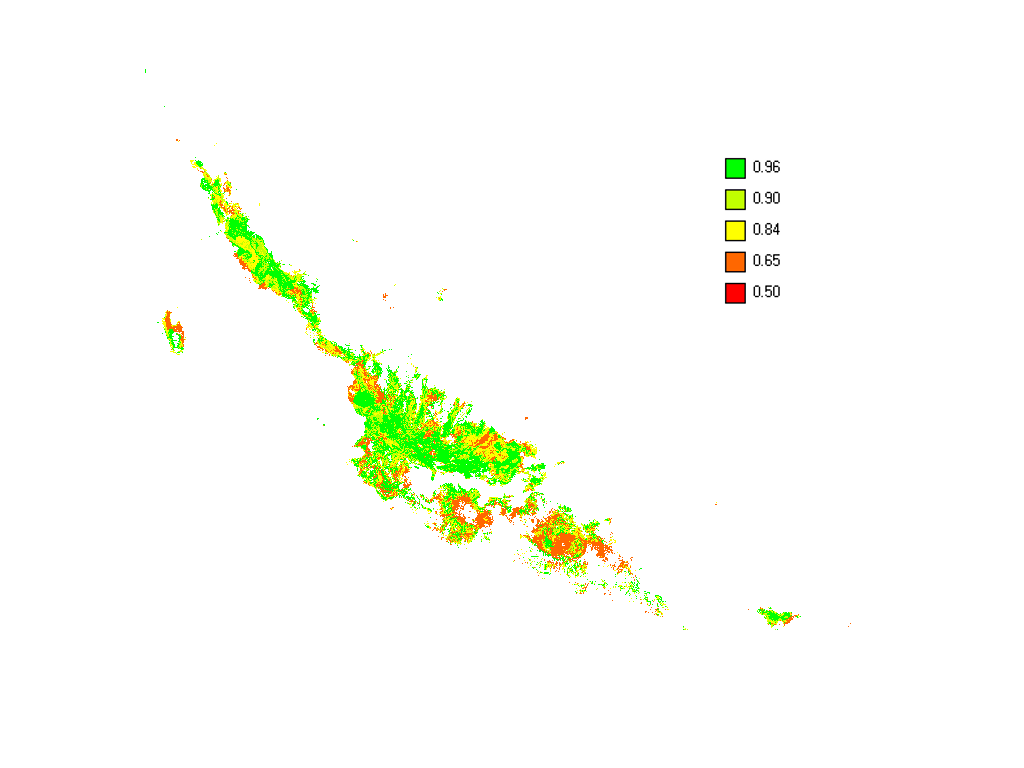
**

**S4.14.** Numerical fuzzy similarity map results for Orr et al. 2014. The fuzzy similarity map and corresponding Fuzzy Kappa statistic, using the ‘Numerical Fuzzy kappa algorithm’, a value of 1.0 (dark green) means total similarity and a value of 0.0 total dissimilarity. Values in red (around 0.50) point to some similarity in both maps.

**
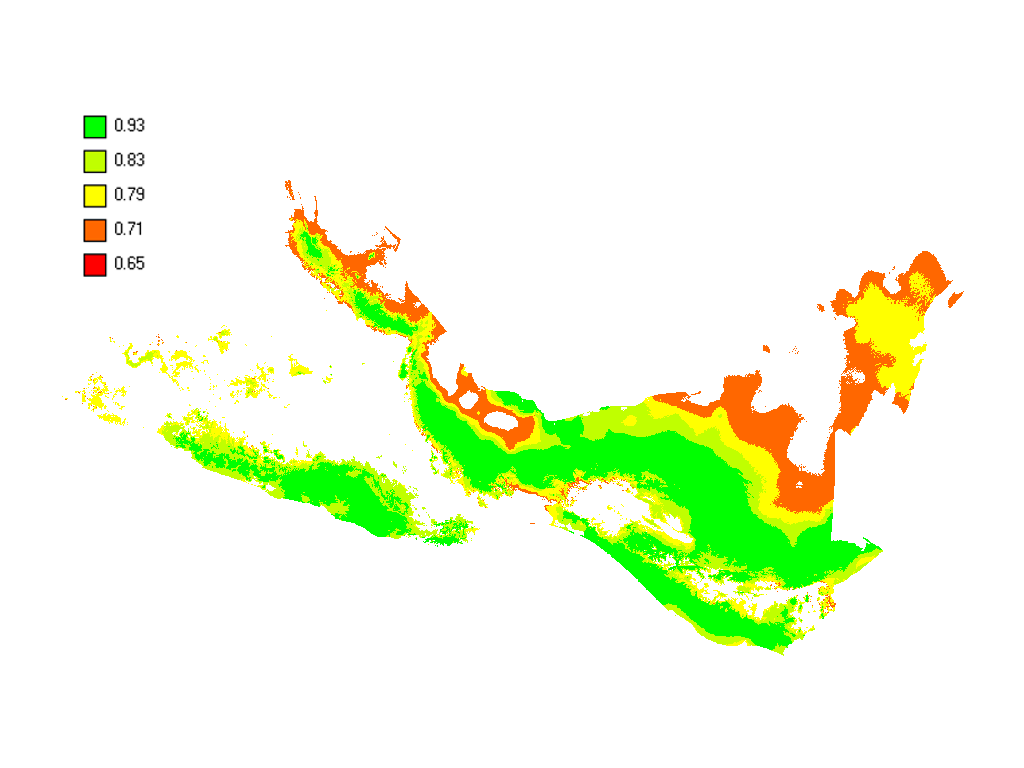
**

**S4.15.** Numerical fuzzy similarity map results for Palma-Perez et al. 2013. The fuzzy similarity map and corresponding Fuzzy Kappa statistic, using the ‘Numerical Fuzzy kappa algorithm’, a value of 1.0 (dark green) means total similarity and a value of 0.0 total dissimilarity. Values in red (around 0.65) point to moderate similarity in both maps.


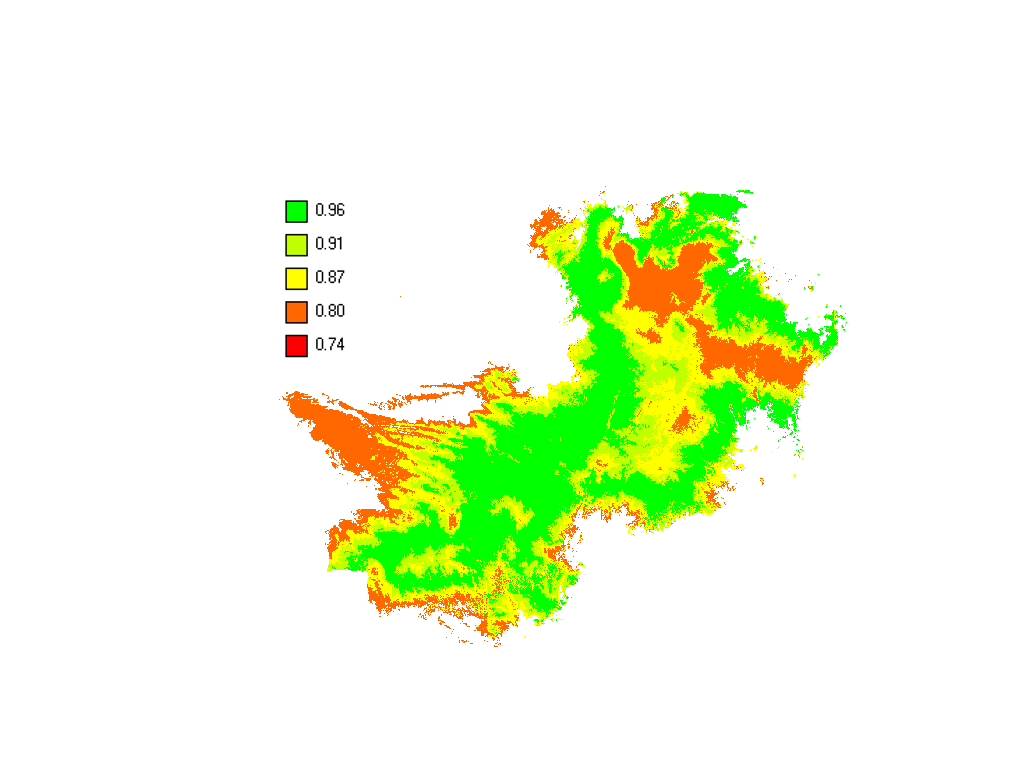


**S4.16.** Numerical fuzzy similarity map results for Pedersen et al. 2014. The fuzzy similarity map and corresponding Fuzzy Kappa statistic, using the ‘Numerical Fuzzy kappa algorithm’, a value of 1.0 (dark green) means total similarity and a value of 0.0 total dissimilarity. Values in red (around 0.65) point to moderate similarity in both maps.

**
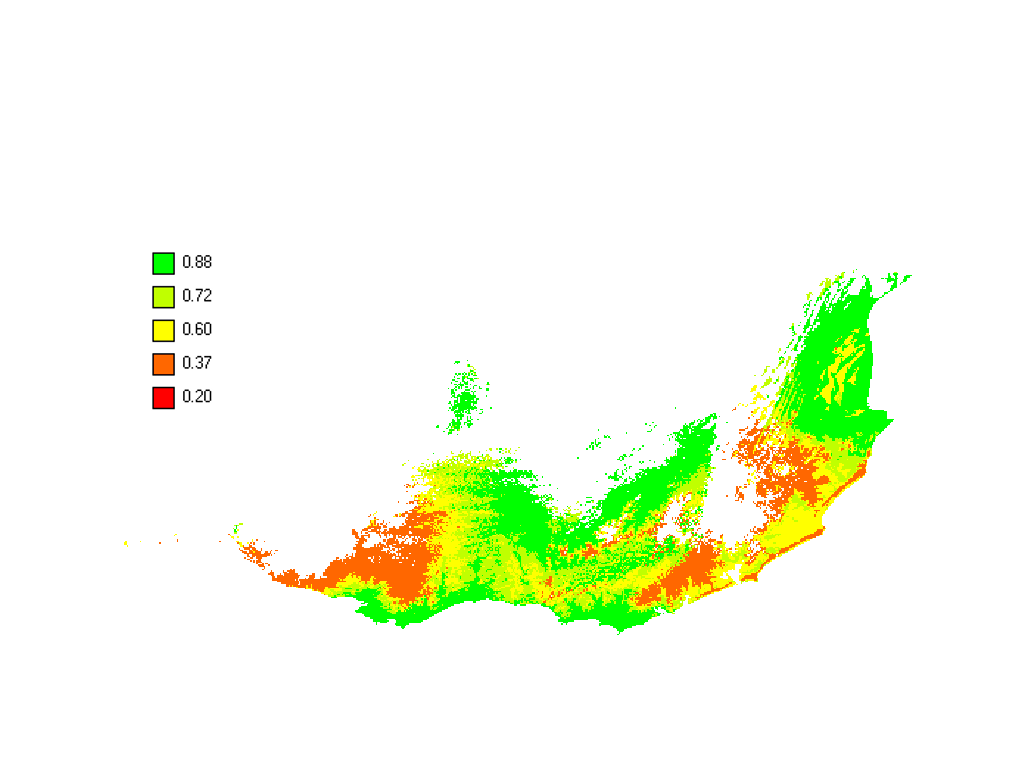
**

**S4.17.** Numerical fuzzy similarity map results for Simo et al. 2014. The fuzzy similarity map and corresponding Fuzzy Kappa statistic, using the ‘Numerical Fuzzy kappa algorithm’, a value of 1.0 (dark green) means total similarity and a value of 0.0 total dissimilarity. Values in red (around 0.20) point to low similarity in both maps.

**
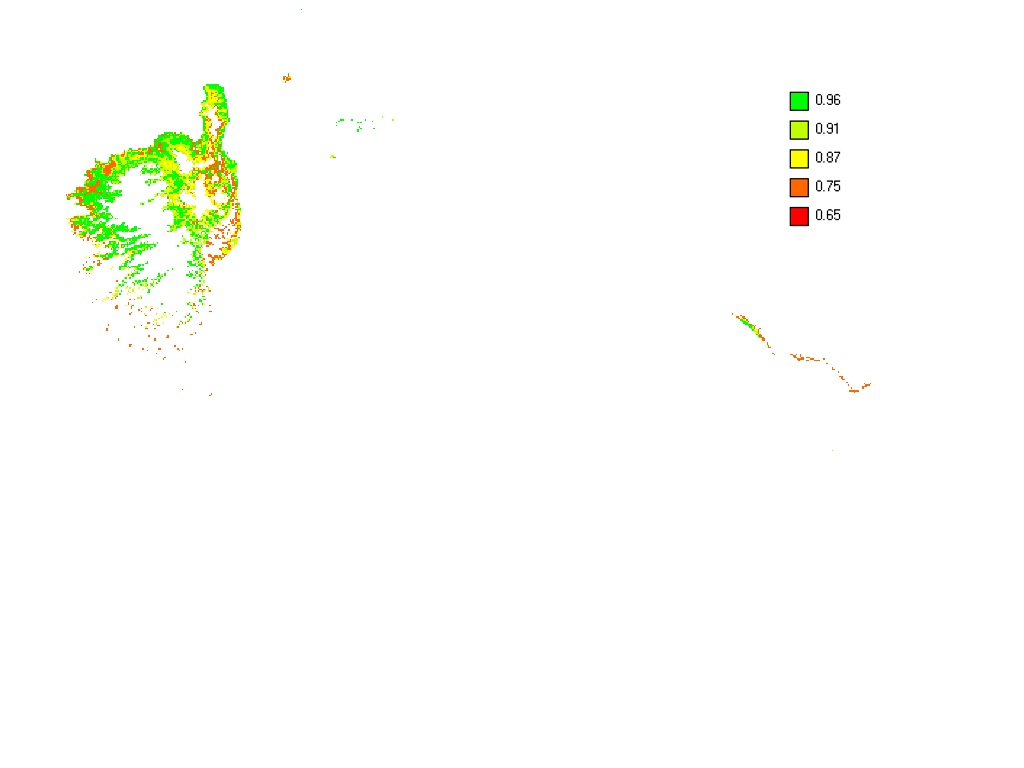
**

**S4.18.** Numerical fuzzy similarity map results for Urbani et al. 2015. The fuzzy similarity map and corresponding Fuzzy Kappa statistic, using the ‘Numerical Fuzzy kappa algorithm’, a value of 1.0 (dark green) means total similarity and a value of 0.0 total dissimilarity. Values in red (around 0.65) point to moderate similarity in both maps.

**
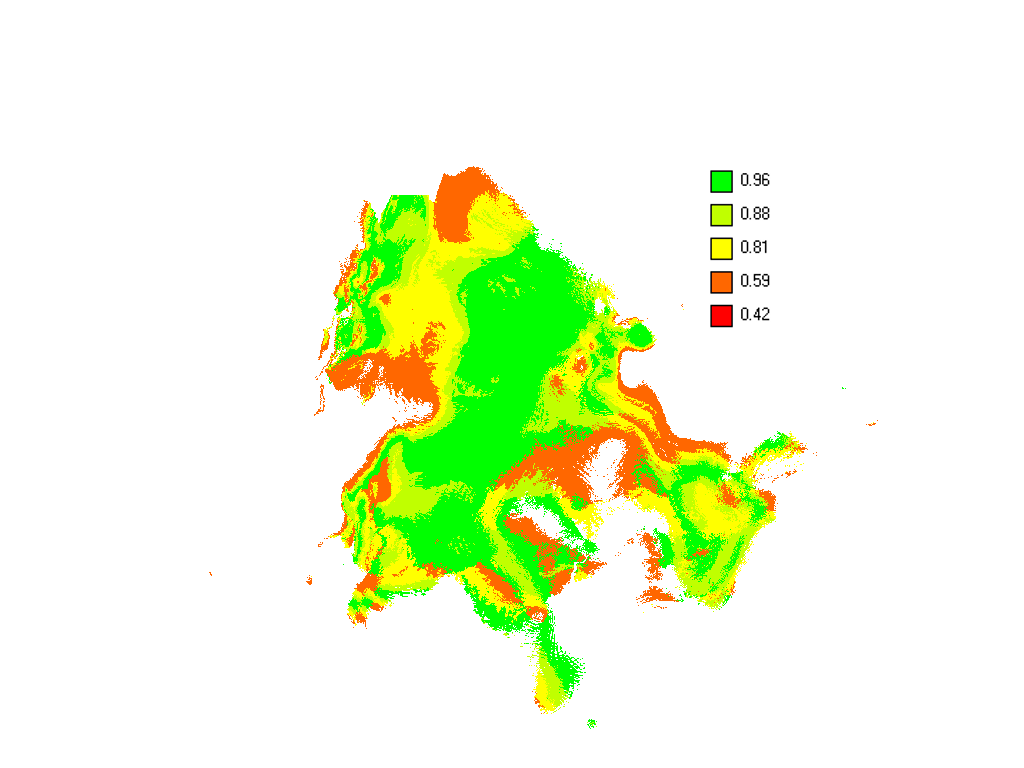
**

**S4.19.** Numerical fuzzy similarity map results for Vergara et al. 2015. The fuzzy similarity map and corresponding Fuzzy Kappa statistic, using the ‘Numerical Fuzzy kappa algorithm’, a value of 1.0 (dark green) means total similarity and a value of 0.0 total dissimilarity. Values in orange (around 0.59) point to moderate similarity in both maps.

**
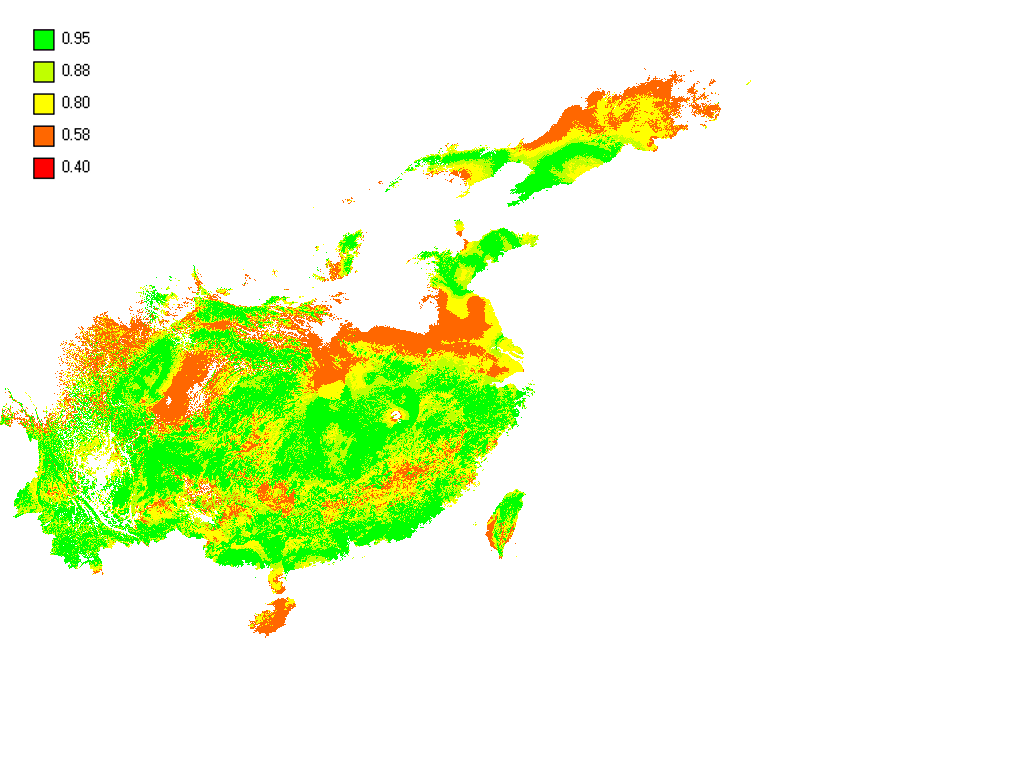
**

**S4.20.** Numerical fuzzy similarity map results for Yu et al. 2013. The fuzzy similarity map and corresponding Fuzzy Kappa statistic, using the ‘Numerical Fuzzy kappa algorithm’, a value of 1.0 (dark green) means total similarity and a value of 0.0 total dissimilarity. Values in orange (around 0.58) point to some similarity in both maps.
